# Presentation and treatment of two massive intrapericardial lipoma. Case report

**DOI:** 10.1016/j.ijscr.2023.108851

**Published:** 2023-09-19

**Authors:** Simone Tombelli, Domenico Viggiano, Alberto Salvicchi, Stefano Bongiolatti, Alessandro Gonfiotti, Luca Voltolini

**Affiliations:** Division of Thoracic Surgery, Careggi University Hospital, Florence, Italy

**Keywords:** Case report, Intrapericardial lipoma, Thoracic surgery

## Abstract

**Introduction and importance:**

Primary pericardial tumors are very rare with an overall incidence of 0.001–0.007 % and account for approximately 10 % of heart neoplasms. We present two clinical cases of massive mature intrapericardial lipomas (maximum size 270 × 230 mm) that were successfully treated in our department.

**Case presentation:**

The first case is that of a 67-year-old male patient who underwent diagnostic investigations after the onset of dyspnea, which confirmed an intrapericardial mass of 270 × 230 mm in size that extended into the left lung field and was treated surgically by a clamshell incision. The second case is that of a 48-year-old patient who was completely asymptomatic and occasionally confirmed to have a 170 × 110 mm intrapericardial mass around the heart, which was surgically removed via sternotomy, also resulting in a mature lipoma.

**Clinical discussion:**

In asymptomatic patients with small lesions, close monitoring is generally indicated. In asymptomatic patients with large lesions the decision should be made after multidisciplinary (MDT) evaluation. In symptomatic patients, surgical treatment is indicated. Lipomas are usually mature lesions with a capsule connected to the origin structure by one or more pedicles. Once reached the cardiac level and opened the pericardium, attention must be paid in resecting these pedicles given the area of origin and the possibility that they may be associated with vital structures.

**Conclusion:**

Both cases were characterized by slow recovery of normal cardiac function in the postoperative course. The average length of patient stay was 12 days, and one case was noted for readmission because a slight increase in pericardial effusion was detected at the scheduled ultrasound check after discharge. After further expert evaluation and steriodine therapy, the patient was discharged to a healthy home after 5 days. This report aims to describe the decision-making process, successful surgical treatment and outcomes of two rare massive intrapericardial tumors.

## Highlights

• Primary pericardial tumors are rare with an overall incidence of 0.001-0.007% and account for ≈10% of cardiac neoplasms.
• We present two clinical cases of massive mature intrapericardial lipoma (maximum size 270x230 mm) treated surgically.
• Once reached the cardiac level and opened the pericardium, attention must be paid in resecting the pedicles.
• Both cases were characterized by slow recovery of normal cardiac function in the postoperative course.

## 1 Introduction

Primary pericardial tumors are very rare with an overall incidence of 0.001–0.007 % and account for approximately 10 % of cardiac neoplasms [1]. The most common benign pericardial tumors are intrapericardial lipomas (accounting for 8.4 % [2]), pericardial cysts, and hemangiomas [3,4]. Lipomas are benign soft tissue tumors consisting of mature fat cells often surrounded by a fibrous capsule [2].

Often these lesions are incidental findings in otherwise asymptomatic patients. When they cause clinical symptoms, it may depend on both the location of the lesion and its mass in relation to adjacent structures [5]. Imaging plays an important role in the detection and definition of intrapericardial tumors. Usually, the diagnostic approach begins with a chest radiograph or echocardiography. CT shows the location of the tumor, its relationship to adjacent structures, and its invasion of vital structures. CT also helps narrow down differential diagnoses, as some of these lesions can be characterized based on their attenuation scores or enhancement pattern. Magnetic resonance imaging (MRI) provides higher contrast resolution compared with CT and is therefore a more powerful tool for lesion characterization [1].

## 2 Description

**Fig. 1:**
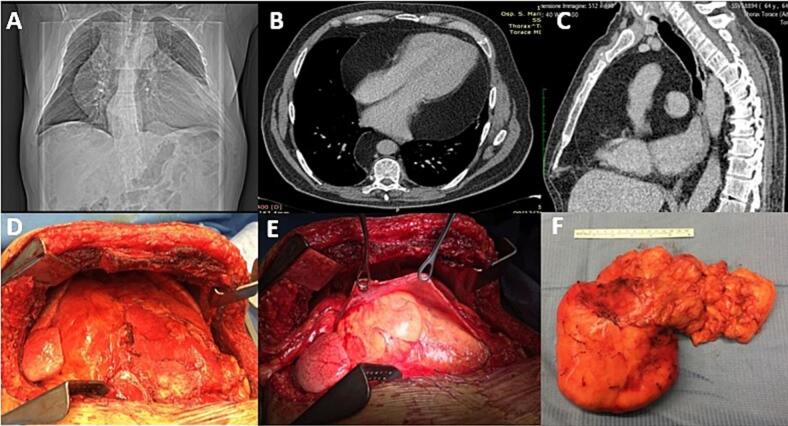
A: Chest Rx; B: thorax CT scan; C: sagittal reconstruction of thorax CT scan; D: view of the lesion after clamshell incision; E: view of the lesion after pericardial incision; F: large lipoid mass extracted from pericardium.

A 67-year-old man was referred to our department (Careggi University Hospital, Florence, Italy) because of progressive dyspnea, reaching level 3 on the mMRC dyspnea scale, and persistent fatigue. On examination, the patient had the following characteristics: BMI 31,1 kg/m^2^, pulse rate 76 bpm, blood pressure of 143/76 mmHg. ECG showed no significant changes. Chest X-ray in anteroposterior view showed significant enlargement of the cardiac silhouette [Fig. 1.A]. Subsequent echocardiogram showed a mass occupying the pericardial space and no other abnormalities. CT Thoracic examination confirmed the presence of a huge intrapericardial homogenous mass measuring 270 × 230 mm that extended into the left lung field, with a marked loss of lung volume and without direct infiltration of the heart and great vessels [Fig. 1.B/C]. The mass had signal intensity similar to fat [Fig. 1.B/C] and there was no associated lymphadenopathy. Pulmonary function tests showed marked impairment of lung volume. Arterial blood gas analysis revealed hypoxemia (PO2 < 65 mmHg) with normal pH. After a complete functional examination the patient was referred for surgical excision of the tumor [Fig. 1.D/E], which confirmed a large mass growing into the pericardial space that was successfully removed after a clamshell incision [Fig. 1.F]. Histopathologic examination of the mass confirmed the diagnosis of benign lipoma.

**Fig. 2:**
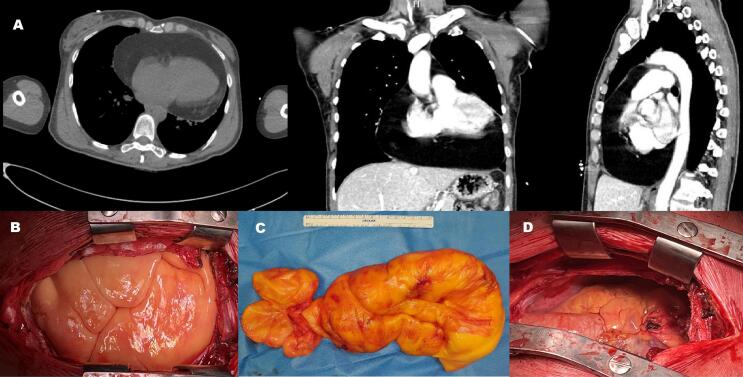
A: Thorax CT scan with sagittal reconstruction; B: view of the lesion after sternotomic incision; C: large lipoid mass extracted from pericardium; D: view of the opened pericardium after mass removal.

The second case was a 48-year-old female patient who was completely asymptomatic and was found to have an occasional mass on a Chest radiography prior to screening colonoscopy. The patient's characteristics were: BMI 19,6 kg/m^2^, pulse rate 59 bpm, blood pressure of 110/65 mmHg. ECG showed no significant changes. After the CT scan, the mass showed homogeneous fat content at the intrapericardial level, completely surrounding the heart to the origin of the epiaortic vessels (maximum diameter 170 × 110 mm) [Fig. 2.A]. No direct infiltration of the heart, great vessel or lungs. Cardiac MRI could not be performed because of severe claustrophobia and inability to perform the examination under anaesthesia. Immunophenotypic typing of peripheral blood revealed no significant qualitative changes in lymphocyte subpopulations. After MDT evaluation, the patient underwent surgical removal of the mass via a sternotomic approach [Fig. 2.B/C]. Histopatologic examination of the mass confirmed the diagnosis of benign lipoma.

## 3 Discussion

Intrapericardial lipomas may occasionally occur in otherwise healthy individuals and therefore do not have a hemodynamic or compressive effect due to the impact of the mass on adjacent structures [2]. When present, symptoms may range from dyspnea, fatigue, palpitations, arrhythmias to syncope and precordial pain [6,7].

In symptomatic patients or those with bulky lesions, it is indicated to perform examinations that may include chest CT, Magnetic Resonance Imaging (MRI), and transthoracic echocardiography (TTE). During the assessment phase, attention should be paid to the location of the tumor and the relationships between it and adjacent structures. The effects of compression on the cardiac chambers and the relationships with the coronary arteries must be carefully studied.

MRI also allows assessment of the density and homogeneity of the tissues composing the lesion and confirm the suspicion of tumors containing fat (lipomas or liposarcomas). Lipomas are characterized by the presence of mature adipose tissue, resulting in a homogeneous density of the structures that make up the lesion. Liposarcomas, on the other hand, are malignant tumors with a lower degree of differentiation that results in a nonuniform density [2,8].

Lipomas should be distinguished from liposarcomas not only by cardiac MRI features or imaging but also histologically.

In asymptomatic patients with small lesions, close monitoring is generally indicated. In asymptomatic patients with large lesions, the decision should be made after multidisciplinary (MDT) evaluation. Key factors in decision making should include: tumor size, location and relationship to intrapericardial structures, growth rate, and ability to differentiate between lipoma and liposarcoma on imaging. The patient's wishes should also be considered. In symptomatic patients, surgical treatment is indicated [3].

In the first case described, we opted for the Clamshell incision, which allowed us to surgically control the mediastinum at any site and to safely work on the entire cardiovascular system because the intrapericardial lipoma completely encircled the heart. However because the intrapericardial lipoma was predominantly anterior, we opted for a median sternotomy in the second case, which provided adequate control of the vascular and cardiac structures.

Lipomas are usually mature lesions with a capsule connected to the originating structure by one or more pedicles. Once we reach the cardiac level and open the pericardium, we must be particularly careful when resecting these pedicles given the area of origin and the possibility that they may form relationships with vital structures [Fig. 2.D]. In cardiothoracic surgery and especially in the removal of large intrapericardial tumors, the role of the anesthesiologist in the assessment and management of the patient's cardiac function in the immediate postoperative period is also essential. Attention must be paid to the patient's water and electrolyte balance, rhythm and cardiac function, the use of medications that may affect cardiac function, and the possible occurrence of pericardial effusion.

Both cases were characterized by a slow recovery of normal cardiac function in the postoperative course, with an episode of atrial fibrillation that returned after pharmacological cardioversion and the occurrence of a pericardial effusion that did not affect the patients' hemodynamics and did not require drainage or pericardiocentesis. The average length of stay of patients was 12 days, and one case was recorded as a readmission because a slight increase in pericardial effusion was detected at the scheduled ultrasound check after discharge. After further expert evaluation and steriodine therapy, the patient was discharged to a healthy home after 5 days. Cardiac follow-up, performed in the second patient about one month after surgery, showed a gradual decrease in pleural effusion and normal cardiac function. The follow-up examination, performed about four months after surgery, showed vital signs in the normal range and normal cardiac function without pericardial effusion in both patients. The next cardiac and radiological follow-up is expected approximately 6 months after the last visit if there is no change in clinic.

## 4 Conclusion

MDT evaluation of each case is essential because massive intrapericardial lipomas are extremely rare lesions and there are many factors that must be evaluated to make the correct treatment decision. After appropriate preoperative evaluation, including CT and an MRI scan when possible, surgery may be indicated in asymptomatic patients if they have massive lesions that are growing rapidly or if imaging is insufficient to differentiate between lipoma and liposarcoma. Surgical procedures should be performed in high-volume centres. Once the surgical indication is made and the lesion removed, coordination with other medical players such as the anesthesiologist and cardiologist is equally important for optimal management of postoperative cardiovascular function.

## Consent

Written informed consent was obtained from the patient for publication of this case report and accompanying images. A copy of the written consent is available for review by the Editor-in-Chief of this journal on request.

## Methods

The work has been reported in line with SCARE criteria [9].

## Ethical approval

This article does not contain any study with human participants or animals performed by any of the authors. Our institutional review board granted approval and waived the requirement for specific informed consent for this case report.

## Funding

The authors declare that the research was conducted in the absence of any commercial or financial relationships.

## Author contribution

Simone Tombelli and Domenico Viggiano contributed to the study concept, acquisition and interpretation of data. Simone Tombelli and Alberto Salvicchi wrote the paper. Alessandro Gonfiotti and Luca Voltolini revising it critically for important content. All authors contributed to manuscript revision, read, and approved the submitted version.

## Guarantor

Simone Tombelli.

## Research registration number

Not applicable.

## Conflict of interest statement

The author declares that he has no relevant or material financial interests that relate to the research described in this paper. No conflicts of interest were shown.
